# Genetic diversity and phylogenetic analysis of Chinese Han and Li ethnic populations from Hainan Island by 30 autosomal insertion/deletion polymorphisms

**DOI:** 10.1080/20961790.2019.1672933

**Published:** 2019-12-13

**Authors:** Jing Liu, Ziwei Ye, Zheng Wang, Xing Zou, Guanglin He, Mengge Wang, Shouyu Wang, Yiping Hou

**Affiliations:** Institute of Forensic Medicine, West China School of Basic Medical Sciences & Forensic Medicine, Sichuan University, Chengdu, China

**Keywords:** Forensic sciences, forensic genetics, InDels, Investigator DIPplex kit, Hainan Han, Hainan Li

## Abstract

With the characteristics of low mutation rate, length variation and short amplicon size, insertion/deletion polymorphisms (InDels) have the advantages of both short tandem repeats (STRs) and single nucleotide polymorphisms (SNPs). Herein, people of two ethnicities from Hainan Island were genotyped for the first time using the Investigator DIPplex kit. We investigated the forensic parameters of the 30 InDels and the phylogenetic relationships among different populations. The accumulated powers of discrimination and powers of exclusion were 0.999 999 999 9646 and 0.9897 in the Hainan Han population and 0.999 999 999 9292 and 0.9861 in the Hainan Li population, respectively. Additionally, population comparisons among geographically, ethnically and linguistically diverse populations *via* cluster heatmap, multidimensional scaling, principal component analysis, phylogenetic tree and STRUCTURE analyses demonstrated that the Hainan Han population had genetic similarities to the other Han, She and Tujia populations, while the Hainan Li population had close genetic relationships to the Zhuang and Miao groups; both populations had a high degree of genetic differentiation from most Turkic-speaking populations. Aforementioned results suggested that the 30 autosomal InDels are highly polymorphic and informative, which are suitable for human identification and population genetics.

## KEYPOINTS

- Four hundred and forty-five Chinese individuals from two ethnicities (Hainan Han and Hainan Li) were firstly analyzed by 30 autosomal InDels included in the Investigator DIPplex panel.
- Forensic parameters of the 30 InDels in the two populations showed high polymorphism and universality for human identification purposes.
- The Investigator DIPplex panel had a certain capacity of differentiating intercontinental populations and different language populations.

## Introduction

Insertion/deletion polymorphisms (InDels), also known as DIPs, are abundant in the human genome and usually appear biallelic feature, which have attracted the interests of forensic researchers and population geneticists recently [1–3]. With the characteristics of low mutation rate, small amplicon size, length variation as well as absence of stutter peaks and so on, InDels combine the advantages of both short tandem repeats (STRs) and single nucleotide polymorphisms (SNPs) markers and are gradually becoming a promising approach in forensic applications (especially for degraded DNA and mixed stain identification), population substructure analysis and biogeographic ancestry inference [2,4–6]. The Investigator DIPplex kit (Qiagen, Valenci, CA, USA), which contains 30 biallelic autosomal InDels and amelogenin, has been validated and studied in some populations to evaluate its efficacy in forensic applications [7–12]. However, the genetic polymorphisms and forensic parameters of the 30 InDels in the Hainan Han and Hainan Li populations remain unknown.

Hainan Island, the second largest island in China, is located at the southernmost tip of China, facing the Mainland to the north across the Qiongzhou Strait (https://en.wikipedia.org/wiki/Hainan). Based on the 2010 National Population Census (http://www.stats.gov.cn/tjsj/pcsj/rkpc/6rp/indexch.htm), Han (84%) and Li (15%) are the dominant ethnicities of Hainan Island and account for 99% of the island’s population. At the time of the Song dynasty (A.D. 960–1279), large numbers of Han people from the Mainland arrived and settled on Hainan Island. However, according to the literature and archeological studies, scholars believe that the Li people are the original inhabitants of Hainan Island and represent descendants of the ancient Yue tribe, who settled on the island between 7 000–27 000 years ago [13,14]. They have their own language, Hlai, which is a subbranch of the Tai-Kadai language family [15]. The special geographic location, population characteristic and history of Hainan Island provide precious resources to conduct genetic-related analysis.

In the present study, we firstly genotyped Han and Li ethnicities from Hainan Island using the Investigator DIPplex kit and evaluated the forensic efficiency of this panel in the aforementioned two populations. Then, we performed population comparisons and genetic structure analysis between the two investigated groups and other previously studied populations based on 30 autosomal InDels. The details of the relevant populations are displayed in Supplementary Table S1.

## Materials and methods

### Sample collection and DNA isolation

A total of 445 blood samples were collected from unrelated individuals (238 Han and 207 Li people) living on Hainan Island with written informed consent. The collection of blood samples was approved by the Ethics Committee of Sichuan University. All participants declared that their ancestors have lived on this island for at least three generations. Genomic DNA was isolated utilizing the PureLink Genomic DNA Mini Kit (Thermo Fisher Scientific, Carlsbad, CA, USA) and quantified using the Nanodrop-2000C (Thermo Fisher Scientific) following the manufacturer’s recommendations. Then, DNA samples were normalized to 1.0 ng/uL and stored at −20 °C until amplification.

### PCR amplification and InDel genotyping

The 30 InDels and amelogenin included in the Investigator DIPplex kit were coamplified on a ProFlex 96-well PCR System (Thermo Fisher Scientific) according to the manufacturer’s protocol. Subsequently, PCR products were separated and detected using the Applied Biosystems 3130 Genetic Analyzer (Thermo Fisher Scientific). Allele allocation was carried out using GeneMapper ID-X v1.5 software. Control DNA 9948 (Qiagen) and ddH_2_O were used as positive and negative controls for each batch of genotyping. Our laboratory has been accredited by the China National Accreditation Service (CNAS) for Conformity Assessment and ISO 17025. The experimental methods and procedures of this study were conducted according to the approved guidelines of Institute of Forensic Medicine, Sichuan University.

### Statistical analysis

Allele frequencies and forensic parameters, including observed heterozygosity (H_o_), expected heterozygosity (H_e_), matching probability (PM), power of discrimination (PD), probability of exclusion (PE) and typical paternity index (TPI), as well as the Hardy-Weinberg equilibrium (HWE) and linkage disequilibrium (LD) tests were assessed using the online tool of STRAF [16]. Subsequently, the population relationships between the two studied groups and other previously studied populations were investigated. The heatmaps of allele frequencies and Nei’s standard genetic distances (*R*_st_) were produced with an online tool Morpheus (https://software.broadinstitute.org/morpheus/). *R*_st_ was calculated based on allele frequencies using the PHYLIP 3.695 package. The *R*_st_ matrix was then used to implement a multidimensional scaling (MDS) plot *via* the SPSS software (IBM SPSS, version 19.0; IBM Corp., Armonk, NY, USA) and construct the neighbour-joining (NJ) tree *via* the MEGA v7.0 software [17]. Allele frequency-based principal component analysis (PCA) was carried out using MVSP v3.22 software [18]. Population structure analysis was performed using STRUCTURE v.2.3.4 software with *K* values spanned from 2 to 7 [19]. Besides, Structure Harvester was used to infer the optimal *K* value, CLUMPP v1.1.2 (Rosenberg Lab, Dallas, TX, USA) and Distruct v.1.1 (Rosenberg Lab) were employed to visualize the population genetic structures.

## Results and discussion

### Allele frequency and forensic parameter analysis

**Figure 1.**
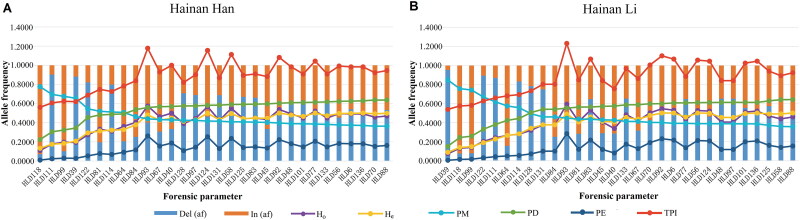
Forensic statistical parameters from 30 InDels in the (A) Hainan Han (*n* = 238) and (B) Hainan Li (*n* = 207) populations. Del: deletion; In: insertion; H_o_: observed heterozygosity; H_e_: expected heterozygosity; PM: matching probability; PD: power of discrimination; PE: probability of exclusion; TPI: typical paternity index.

Supplementary Table S2 presents the genotype data of the 30 InDels in the Hainan Han and Hainan Li populations. No significant deviation from HWE was observed in either population after applying the Bonferroni correction (*P* < 0.0017) (Supplementary Table S3). LD test were performed using a shuffling test for all of the locus by locus pairwise combinations. No significant LD was found in either group, with the exception of HLD93–HLD97 in Hainan Han people after applying the Bonferroni correction (*P* < 0.0001) (Supplementary Tables S4–S5). The observed significance may represent substructure association, which may be caused by the sample size or genetic admixture [20]. The allele frequencies and forensic parameters of the 30 InDels are shown in Supplementary Table S3 and Figure 1. The frequencies of deletion allele varied from 0.0714 to 0.9034 in Hainan Han people and from 0.0749 to 0.9541 in Hainan Li people. The H_o_ ranged from 0.1092 to 0.5756 in Hainan Han people and from 0.0725 to 0.5942 in Hainan Li people. In both populations, HLD88 possessed the largest PD and the values were 0.6361 (Hainan Han) and 0.6423 (Hainan Li), HLD93 had the highest PE, and the values were 0.2627 (Hainan Han) and 0.2840 (Hainan Li). The combined powers of discrimination and exclusion were 0.999 999 999 9646 and 0.9897 in Hainan Han people and 0.999 999 999 9292 and 0.9861 in Hainan Li people. These results suggested that this panel was effective for personal identification but not sufficiently powerful to perform a paternity test in the two groups.

### Genetic differentiation along continental divisions

To gain an overview of the genetic similarities and differences between our studied populations and 56 global populations (East Asian [8–12,21–35], West Asian [36,37], European [33,37–42], American [43–45], African [23,46]), we conducted population comparisons along continental divisions through a series of analyses. In the cluster heatmap (Supplementary Figure S1), the HLD101, HLD114, HLD39, HLD48 and HLD111 loci showed high deletion frequencies in most East Asian populations, while the HLD118, HLD99, HLD64 and HLD81 loci showed low deletion frequencies in these groups. Moreover, three African populations (Xhosa, Zulu and Nigeria groups) displayed high deletion frequencies at the HLD06, HLD124, HLD77, HLD125, HLD58, HLD40 and HLD45 loci and low deletion frequencies at the HLD101, HLD114, HLD128, HLD131, HLD39 and HLD70 loci. In brief, there were significant differences in allele frequency distribution between populations from different continents (especially between East Asian and African populations).

As shown in Supplementary Table S6 and Supplementary Figure S2, the Hainan Han population had the closest relationship with the Shanghai Han population (*R*_st_ = 0.0024), while the Hainan Li population had the smallest *R*_st_ with the Vietnam group (*R*_st_ = 0.0040). Both showed the highest *R*_st_ with the African Nigeria population, and the values were 0.1878 (Hainan Han) and 0.2059 (Hainan Li), respectively. As demonstrated in Supplementary Figure S3(A), our studied populations and most East Asian populations were located at the right part of the *y*-axis, West Asian and European populations were distributed in the second quadrant, and most American and African populations were scattered in the third quadrant. A PCA plot (Supplementary Figure S3(B)) showed that the population distribution pattern was similar to the MDS, and the top two components could explain 74.01% of the total variance (PC1: 55.85% and PC2: 18.16%). Our studied populations and the East Asian populations were separated from other continental populations and had the greatest genetic differences from the three African populations (Xhosa, Zulu and Nigeria groups). Furthermore, the six Xinjiang minorities were situated between the other East Asian and European populations, in accordance with our previous study [47]. As shown in the NJ cladogram (Supplementary Figure S3(C)), three main branches were observed. The lower branch included Brazil and four African populations, the middle one contained six American groups, and the upper one was split into two subclades. Our studied populations and the East Asian populations clustered in the upper subclade, while the lower subclade consisted of the West Asian, European, Afrikaner and Southern Brazil populations. To sum up, the results of MDS, PCA and phylogenetic tree were consistent with each other.

### Genetic differentiation along ethnic and linguistic divisions

For the purpose of obtaining a clearer insight into Chinese population relationships, we performed various analyses among our studied populations and 28 Chinese populations [8–12,21,22,24–33]. Supplementary Figure S4 presents the cluster heatmap of deletion allele frequencies among the 30 populations, and three primary clusters (I, II and III) were easily distinguished. Cluster I was divided into two subclusters (IA and IB); the IA subcluster included five Tibetan groups, and the IB subcluster consisted of the Han, Bai, Tujia, Hui, Xibe, Dongxiang, She, Salar and Liangshan Yi populations. Cluster II contained the Hainan Li, Gaoshan, Zhuang, Miao, Dong and Yunnan Yi populations. In cluster III, there were six Xinjiang minorities. The Hainan Han population had a similar frequency pattern to the other Han, Hui and Tujia populations, while the Hainan Li population had a similar frequency pattern to the Gaoshan, Zhuang and Miao populations.

**Figure 2.**
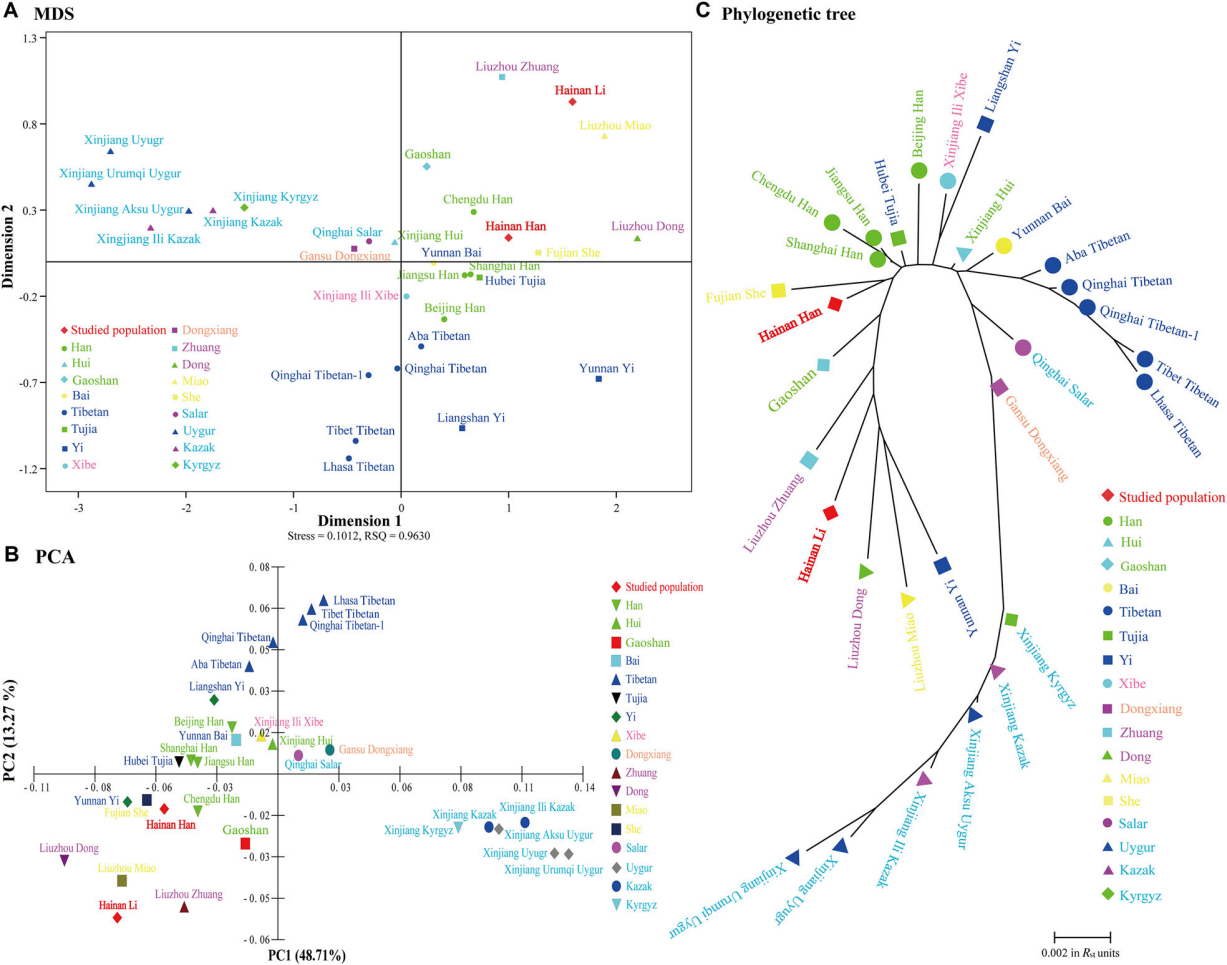
Genetic differentiation along ethnic and linguistic divisions. (A) The multidimensional scaling (MDS) plot was drawn based on pairwise *R*_st_ values. (B) The principal component analysis (PCA) plot was performed based on allele frequencies of 30 InDels. (C) The phylogenetic tree was constructed based on pairwise *R*_st_ values with the NJ method. Different coloured fonts represent different language families (Green: Sinitic; Dark blue: Tibeto-Burman; Pink: Tungusic; Orange: Mongolic; Red purple: Tai-Kadai; Yellow: Hmong-Mien; Light blue: Turkic).

As shown in Supplementary Table S6 and Supplementary Figure S5, the Hainan Han population had relatively small *R*_st_ with the other Han, Hui and Tujia populations, while the Hainan Li population had genetic affinities with the Zhuang, Dong and Gaoshan populations. Both had distant genetic relationships with the six Xinjiang groups. In Figure 2, different coloured fonts represent different language families. The MDS plot (Figure 2A) showed that all of the Turkic-speaking populations were distributed in the second quadrant, and all of the Tibeto-Burman-speaking populations (except the Bai group) were scattered below the *x*-axis. The Hainan Han population clustered with the Tujia, Bai, She (Hmong-Mien-speaking population) and Sinitic-speaking populations, while the Hainan Li population had close genetic relationships with the Zhuang (Tai-Kadai-speaking population) and Miao (Hmong-Mien-speaking population) groups. In our PCA plot (Figure 2B), the first two components accounted for 61.98% of the total variance. Most Turkic-speaking populations were distributed in the fourth quadrant, and most Tibeto-Burman-speaking populations were situated above the *x*-axis. The Hainan Han population had genetic similarities to the She, Tujia, Bai, Yunnan Yi and Sinitic-speaking populations, while the Hainan Li population had genetic affinities with two Tai-Kadai-speaking populations (Zhuang and Dong groups) and the Miao group. In the NJ tree (Figure 2C), three clades were observed: a Turkic-speaking and Mongolic-speaking clade, a Tibeto-Burman-speaking clade (excluding the Yi and Tujia groups), and a clade containing our studied populations and other linguistic groups. The Hainan Han population clustered with the She people and then with three additional Han populations. The Hainan Li population first grouped with the Yunnan Yi, Miao and Dong groups and then with the Zhuang people. In short, we verified the genetic similarities among the same language families through multiple analyses and observed close genetic relationships between the Tai-Kadai-speaking and Hmong-Mien-speaking populations.

### Population structure analysis

**Figure 3.**
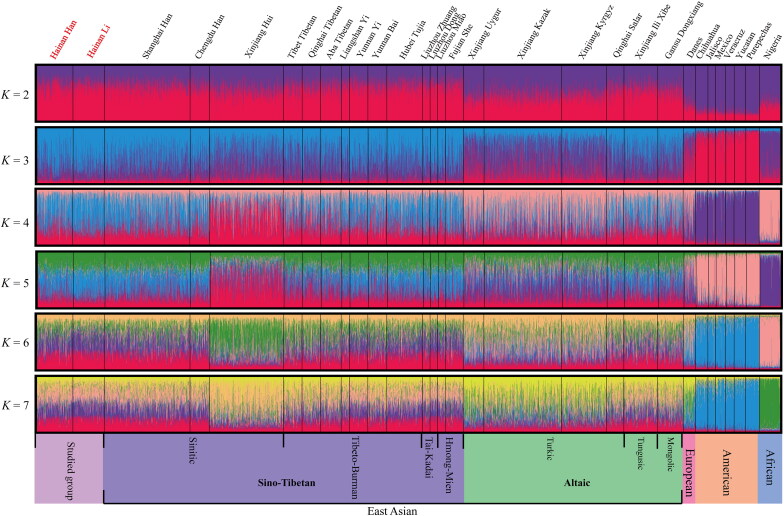
Structure clustering analysis was performed based on genotypes of 30 InDels among 30 populations, assuming *K* = 2–7.

Structure analysis among four continental populations [9–12,22–27,29–32,38,45] is displayed in Figure 3. At *K* = 3, the genetic makeup showed a clear distinction between the East Asian populations and the populations from the other three continents. Our studied populations shared a resemblance to the East Asian groups, apart from the three Turkic-speaking populations. At *K* > 3, no further substructure was distinguished with the exception of the Xinjiang Hui population, which may be caused by the complex history of that group.

## Conclusion

We firstly reported a batch of genotype data of 30 InDels included in the Investigator DIPplex kit in the Hainan Han and Hainan Li groups and evaluated the forensic application ability of this panel in the two populations. Furthermore, population comparisons along geographical, ethnical and linguistic divisions through multiple analyses manifested that the 30-InDels panel had a certain intercontinental differentiation ability (especially between the East Asian and African populations) and could also distinguish different language populations to a certain extent (especially for most Turkic-speaking populations). For our investigated populations, the Hainan Han population had close relationships to the other Han, She and Tujia populations, and the Hainan Li population had genetic affinities with the Zhuang and Miao minorities.

## Supplementary Material

Supplemental Material
