# Supplementary figures and images for: Genetic diversity and phylogenetic analysis of Chinese Han and Li ethnic populations from Hainan Island by 30 autosomal insertion/deletion polymorphisms
Source: Forensic Sci Res. 2019 Dec 13;7(2):189–95. doi: 10.1080/20961790.2019.1672933 (PMC9245983; doi:10.1080/20961790.2019.1672933)

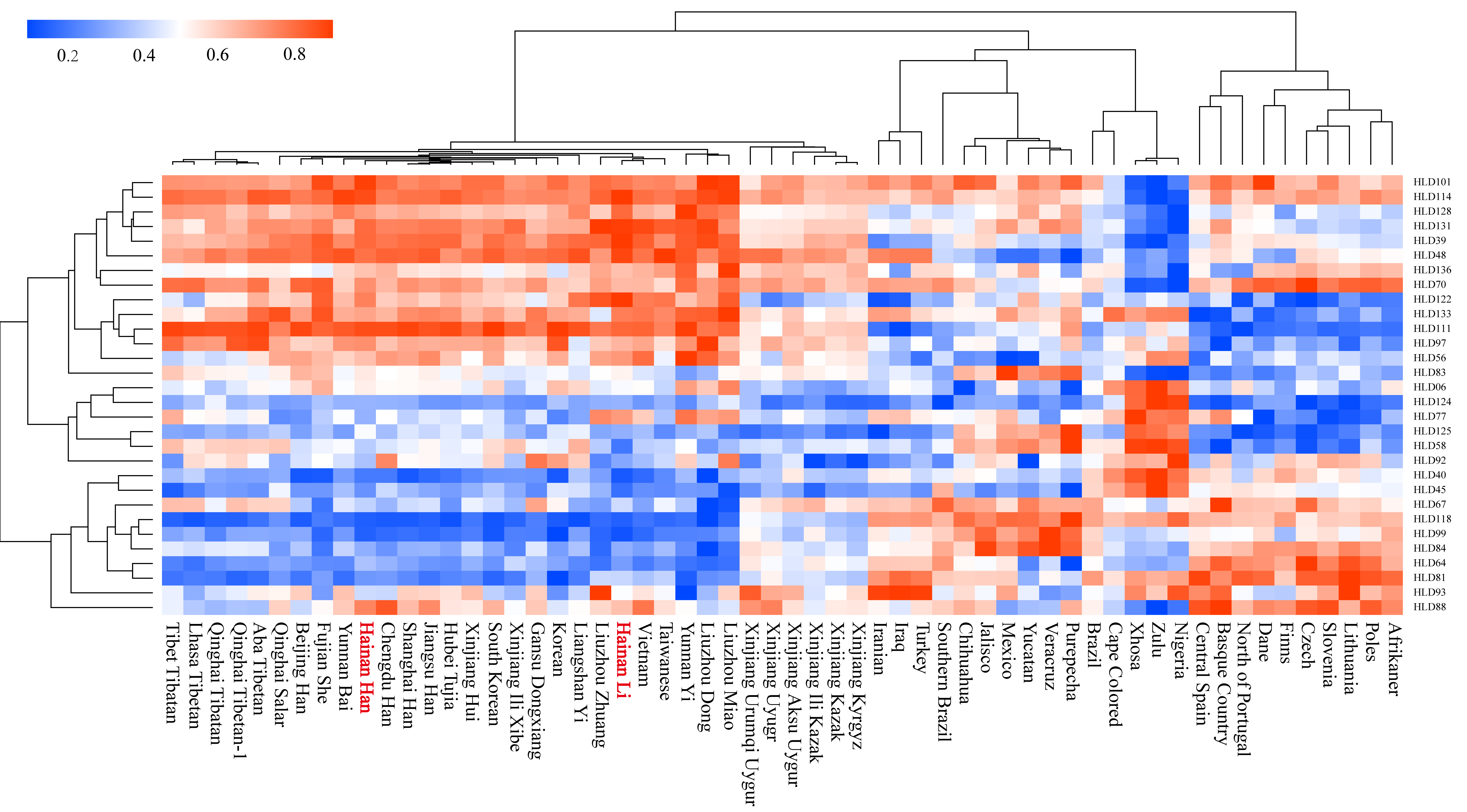

Supplement: Supplemental Material [file TFSR_A_1672933_SM5167.zip › Supplementary Figure S1.tif]

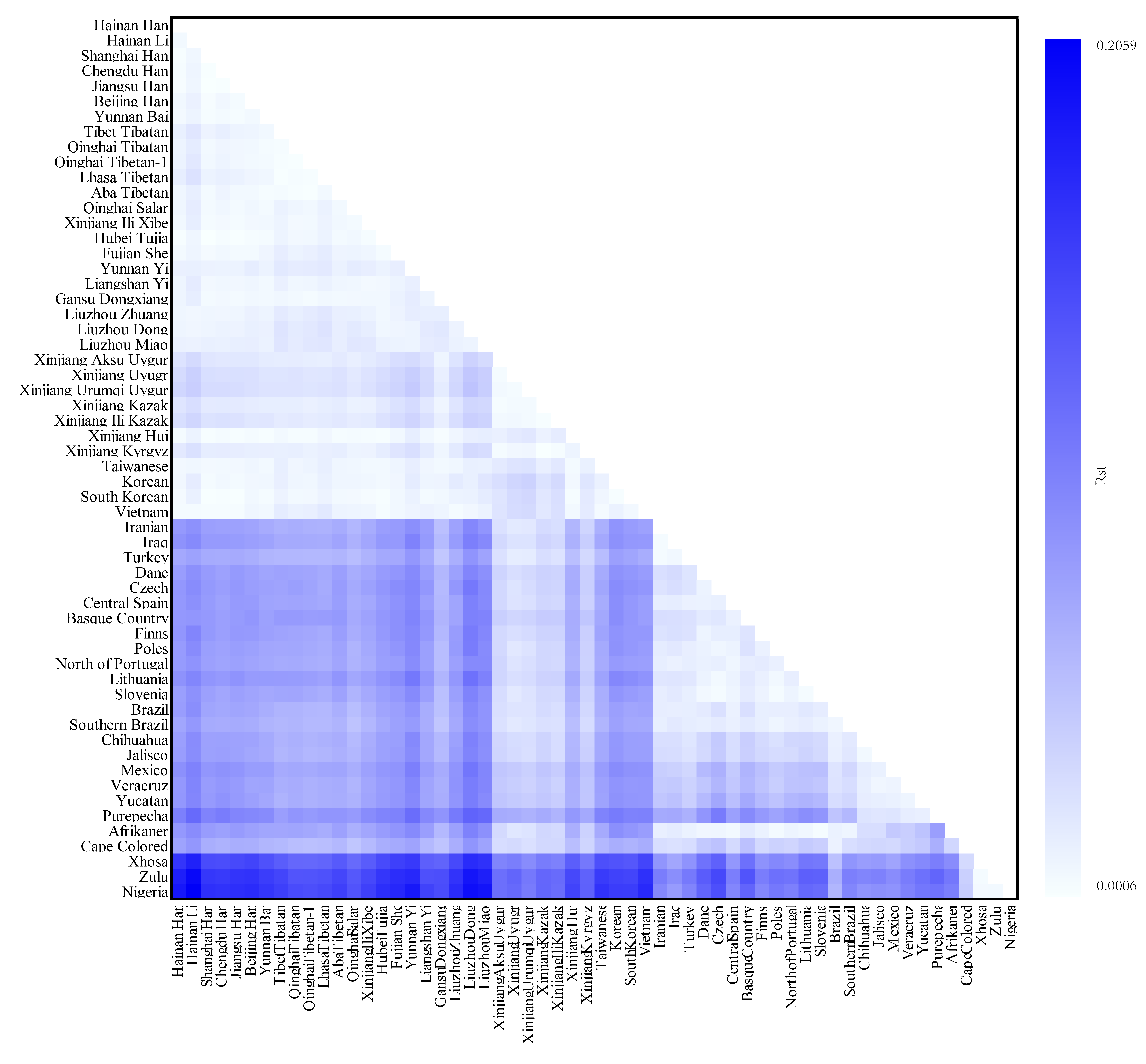

Supplement: Supplemental Material [file TFSR_A_1672933_SM5167.zip › Supplementary Figure S2.tif]

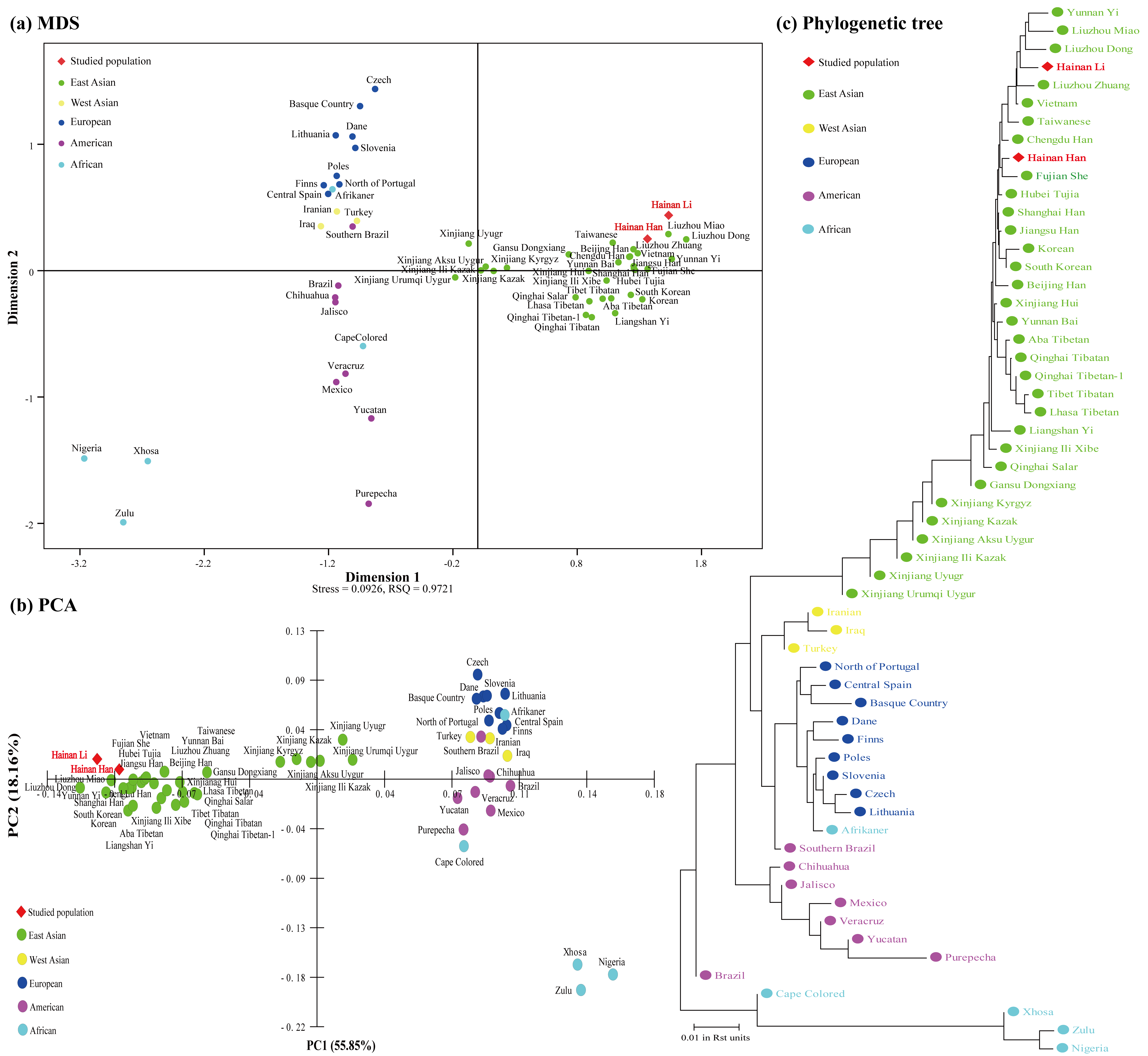

Supplement: Supplemental Material [file TFSR_A_1672933_SM5167.zip › Supplementary Figure S3.tif]

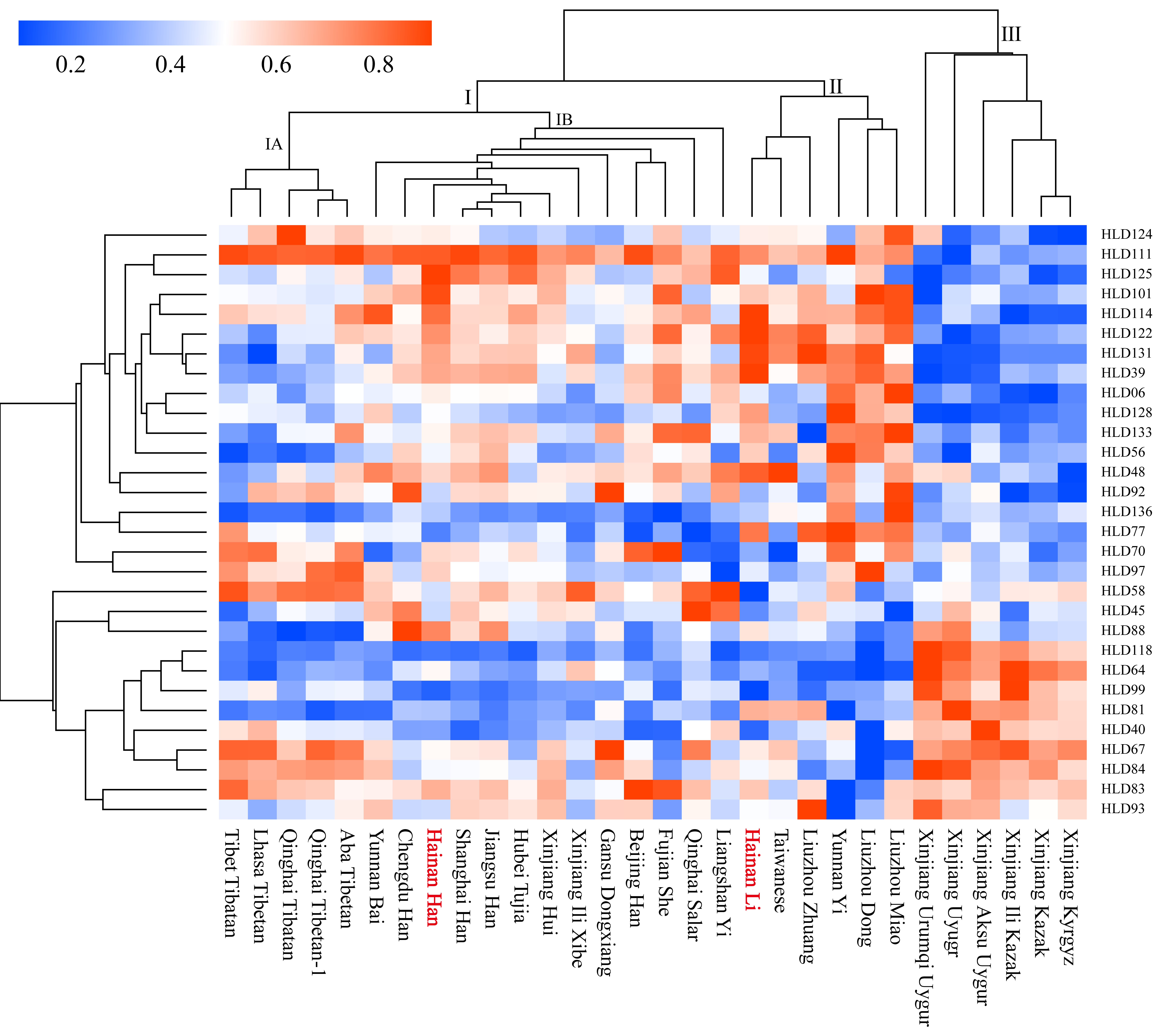

Supplement: Supplemental Material [file TFSR_A_1672933_SM5167.zip › Supplementary Figure S4.tif]

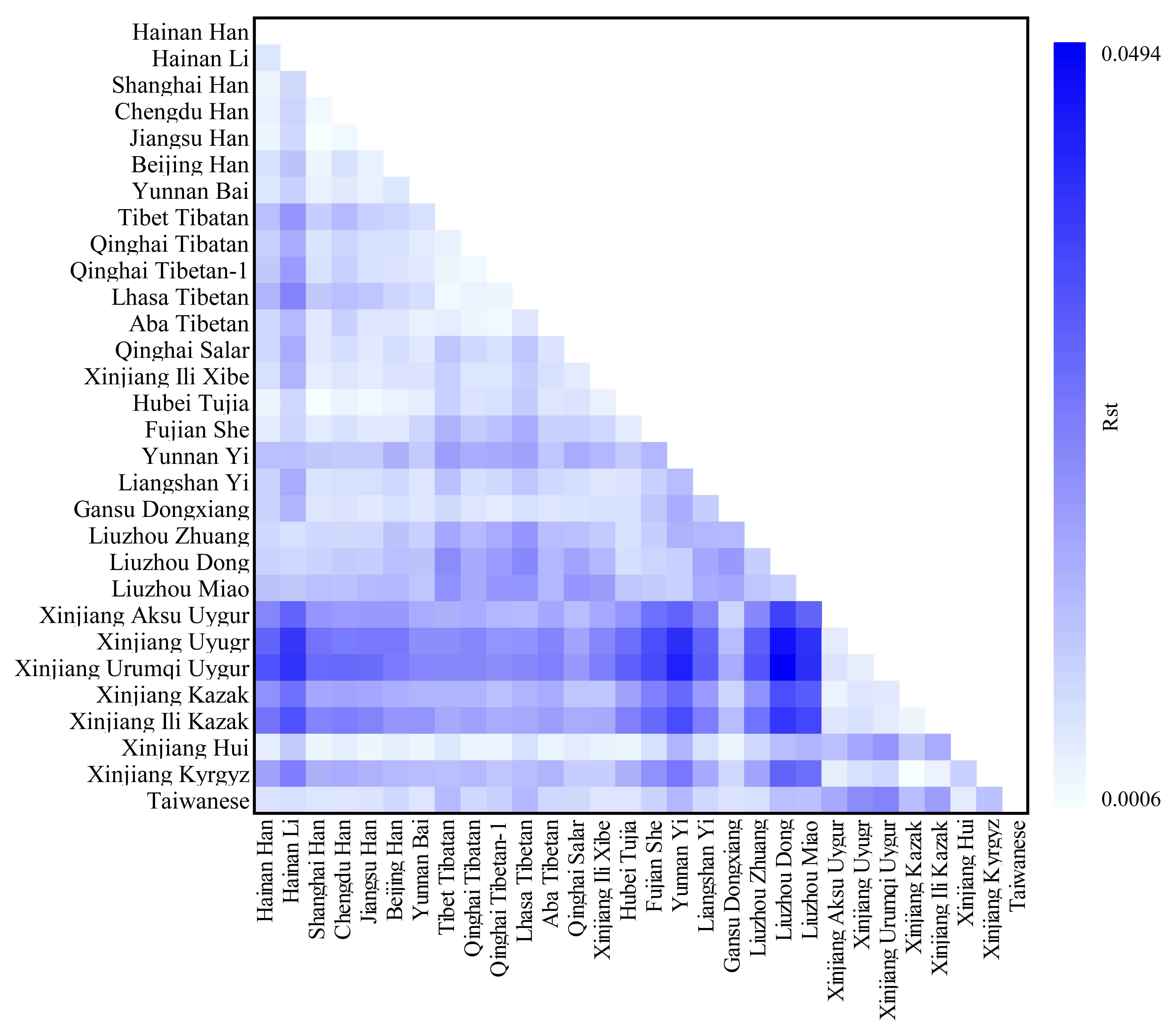

Supplement: Supplemental Material [file TFSR_A_1672933_SM5167.zip › Supplementary Figure S5.tif]
